# The PP2A phosphatase regulatory subunit SsRts1 regulates morphogenesis, mitosis, sexual mating and virulence in *Sporisorium scitamineum*

**DOI:** 10.1080/21505594.2026.2723679

**Published:** 2026-08-23

**Authors:** Jinfeng Qiu, Haoming Wu, Ying Liu, Yu Liu, Lijiu Zhao, Ru Li

**Affiliations:** a Guangxi Sugarcane Bio-Breeding Laboratory, State Key Laboratory for Conservation and Utilization of Subtropical Agro-Bioresources, College of Life Science and Technology, Guangxi University, Nanning, China; b Guangxi Key Laboratory of Sugarcane Biology, College of Agriculture, Guangxi University, Nanning, China

**Keywords:** PP2A regulatory subunit, SsRts1, mitosis, sexual mating, pathogenicity, *sporisorium scitamineum*

## Abstract

Sugarcane smut, caused by *S. scitamineum*, results in substantial economic losses across global sugarcane growing areas. Elucidating the pathogenic mechanisms of *S. scitamineum* is therefore crucial for developing effective disease control strategies. Our previous work has shown that the protein phosphatase SsPpe1 regulates mating and pathogenicity in *S. scitamineum*. In this study, we identified SsRts1 as a novel interacting protein of SsPpe1, which contains a PP2A regulator subunit B56 domain. Deletion of *Ssrts1* gene impaired sporidia growth, resulting in pseudohyphal sporidia with multiple nuclei and aberrant chitin distribution. Further analysis revealed elevated phosphorylation levels of the cell cycle-related kinase CDK1^T161^ in the Δ*Ssrts1* mutants, suggesting that SsRts1 participates in mitosis regulation. Moreover, the Δ*Ssrts1* mutants exhibited increased sensitivity to cell wall, oxidative and high osmotic stresses. Mechanistically, SsRts1 negatively regulates the phosphorylation levels of the CWI-MAPK Mps1, implicating that SsRts1 is involved in regulating the cell wall integrity (CWI) pathway. Additionally, deletion of *Ssrts1* also led to abnormal accumulation of autophagosome, along with defects in sexual mating, filamentation, and virulence. Global transcriptome profiling indicated that SsRts1 modulates ATPase activity, cell cycle progression, transmembrane transport, and tryptophan metabolism. Notably, exogenous supplementation with tryptophan partially restored the mating and filamentation defects in the Δ*Ssrts1* mutants, indicating that SsRts1 modulates these processes partly through the tryptophan catabolism pathway. Collectively, these findings provide insight into the regulatory roles of PP2A regulatory subunit SsRts1 in *S. scitamineum*, and expand our understanding of PP2A function in plant-pathogenic fungi.

## Introduction

Sugarcane is one of the important commercial crops cultivated across more than 90 countries [1]. It is the main supplier of world sugar, accounting for 80% of global sugar production [2]. In addition, it is also a viable source for bioenergy production and several other economically important byproducts. For example, sugarcane is a promising feedstock for bioethanol production due to its high biomass index [3]. Furthermore, sugarcane can be used as microbial fuel to provide energy value [4]. Sugarcane smut, caused by *Sporisorium scitamineum*, is one of the major fungal diseases that significantly reduces sugarcane production and quality. *S. scitamineum* is a typical dimorphic fungus with three distinct forms: haploid yeast-like sporidia, dikaryotic hyphae and teliospore [5]. Haploid yeast-like sporidia cannot infect sugarcane. Following the sexual mating of two opposite sporidia mating types, dikaryotic hyphae are capable of infecting sugarcane. The infected sugarcane will develop a “black whip” composed of sugarcane stem, fungal hyphae, and teliospore [6]. Wind-dispersed teliospores can spread the pathogen to more extensive sugarcane-growing regions, resulting in wider dissemination of sugarcane smut. Infected sugarcane plants typically exhibit stunted growth and produce thin, slender canes with widely spaced nodes, often accompanied by a whip-like sorus at the apex of affected stalks, resulting in substantial reductions in both yield and sugar content [7]. Protein phosphorylation, a reversible post-translational modification (PTM), plays important roles in diverse cellular processes, such as cell cycle regulation, signal transduction, cell metabolism, filamentation, and virulence [8]. In *S. scitamineum*, kinases are critical for stress tolerance, mating/filamentation, and virulence [9–11]. For example, deletion of the genes encoding of the MAP kinase SsKpp2 and the Ser/Thr kinase SsAgc1 results in sexual mating defect in *S. scitamineum* [9,12]. Histidine kinase SsSln1 and cAMP-PKA signaling pathways antagonistically regulated the transcription of pheromone-responsive transcription factor SsPrf1, thereby influencing *S. scitamineum* mating and virulence [59]. Recent studies indicated that the MAPK SsHog1 regulates the oxidative stress response specifically through cytochrome P450 pathway [60]. Protein phosphatases function as molecular switches that modulate the phosphorylation status of target proteins, a process essential for the regulation of various cellular activities [60]. Previous studies revealed that tyrosine phosphatase SsPtp2 mediates dephosphorylation of SsHog1, and its loss leads to impaired mating capacity and attenuated virulence in *S. scitamineum* [59]. However, information about other protein phosphatases and their regulatory roles in *S. scitamineum* remains uncharacterized.

Type 2A protein phosphatase (PP2A), a serine/threonine phosphatase, consists of a scaffold subunit (A), a regulatory subunit (B) and a catalytic subunit (C) [13]. PP2A complex activities and substrate specificity are regulated by the binding of PP2A-B to the core enzyme [14]. Regulatory subunits can interact with various catalytic subunits, thereby mediating the dephosphorylation of substrate proteins. Accordingly, regulatory subunits exhibit more diverse biological functions than catalytic subunits. Based on sequence homology, PP2A-B can be classified into B (B55), B′(B56) and B″ families [15]. In *Arabidopsis thaliana*, the PP2A-B′ γ regulates resistance to *Botrytis cinerea* and leaf senescence [16]. In *Cryptococcus neoformans*, deletion of PP2A-B *far8* impairs growth, cell morphology, virulence, mating, and sexual differentiation, including the production of hyphae, basidia and basidiospore [17]. In *Neurospora crassa*, the PP2A regulatory subunits rgb-1 and b56 are involved in hyphal growth [18]. We recently reported that a PP2A-like phosphatase, SsPpe1, plays an essential role in sexual mating/filamentation and virulence in *S. scitamineum* [19]. However, the regulatory subunit of SsPpe1 and its function in *S. scitamineum* remains unclear. Elucidating this issue is of great significance for better understanding the regulatory mechanisms of protein phosphatases in *S. scitamineum*.

In this study, we identified the SsRts1 protein as an interactor of SsPpe1. Analysis of the protein sequence showed that SsRts1, containing a conserved B56 domain, acts as a PP2A-B′ regulatory subunit. Further results demonstrated that the deletion of *Ssrts1* resulted in defects in sporidia growth, morphogenesis, mitosis, stress tolerance, autophagy, mating/filamentation, and virulence. RNA sequencing (RNA-seq) analysis revealed that SsRts1 is involved in regulating ATPase activity, cell cycle, transmembrane transport and tryptophan metabolism pathway. Collectively, these findings advance our understanding of the PP2A regulatory subunit’s functions in sporidia morphology, mitosis, stress tolerance, autophagy, sexual mating and virulence of *S. scitamineum*.

## Results

### SsRts1 physically interacts with SsPpe1

**Figure 1.**
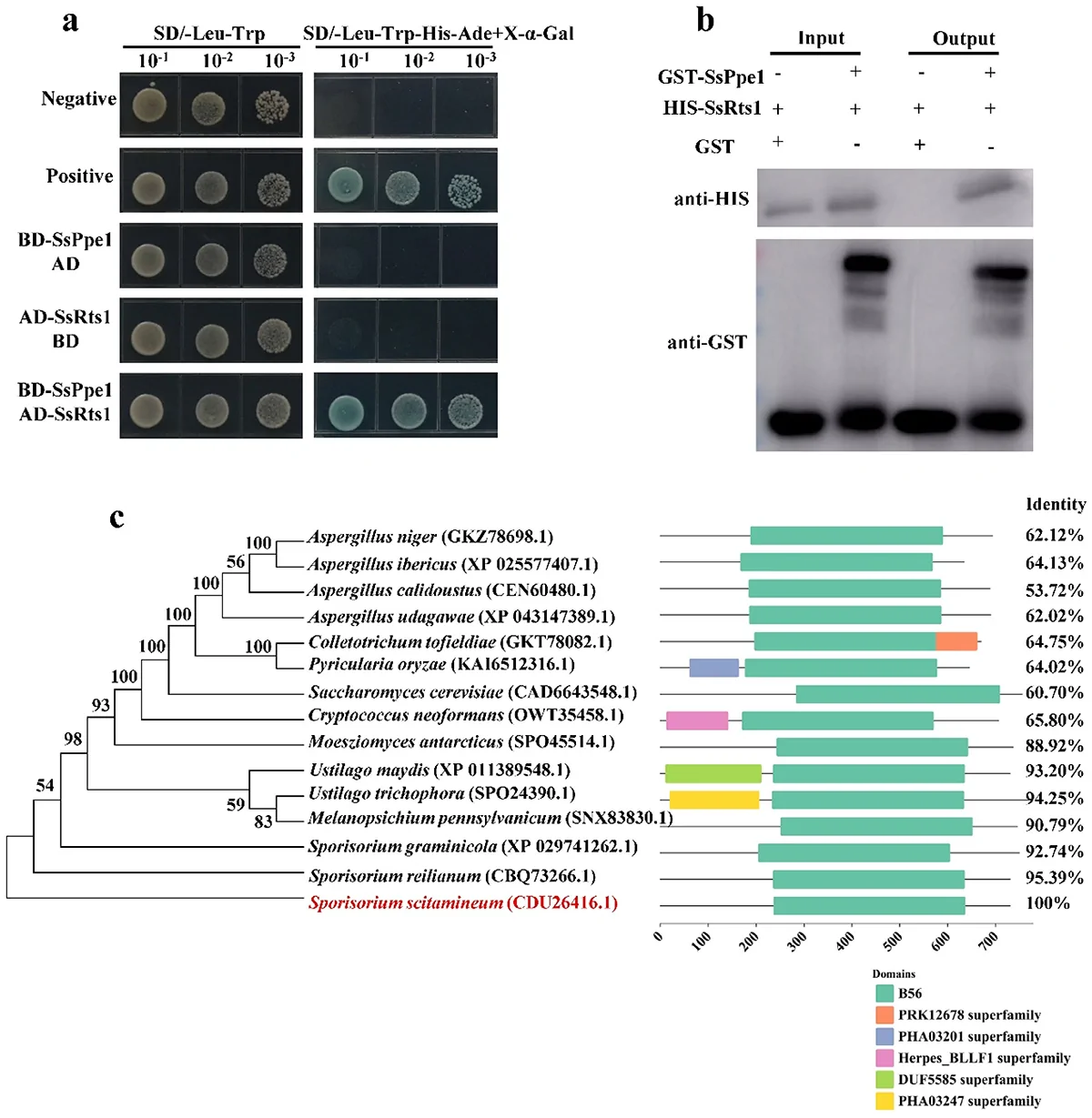
SsRts1 physically interacts with SsPpe1. (a) Y2H analysis of the interaction between SsRts1 and SsPpe1. The pGADT7-T plasmid was transformed with pGBKT7-53 and pGBKT7-lam into yeast cells as the positive and negative controls. BD, pGBKT7; AD, pGADT7. Representative images from three independent biological replicates were shown. (b) GST pull-down assay shows interaction between SsRts1 and SsPpe1. His-SsRts1 was pulled down by GST-SsPpe1 immobilized on GST-tag purification Resin, but not by GST. The input and output proteins were detected with anti-His and anti-GST antibodies. Representative images from three independent biological replicates were shown. (c) Sequence alignment of SsRts1 proteins from*S. scitamineum*and other filamentous phytopathogenic fungi. The phylogenetic tree was constructed using the neighbor-joining method in MEGA 7.0 software. The structural domains of these sequences were analyzed using the NCBI website and the conserved domain plot tool ChiPlot.

Previously, we reported that the PP2A-like phosphatase SsPpe1 plays a role in regulating MAPK signaling pathways, which are critical for mating/filamentation and virulence in *S. scitamineum* [19]. To investigate the underlying regulatory mechanisms, we conducted a yeast two-hybrid (Y2H) screen using a cDNA library constructed from total RNA isolated from *S. scitamineum* to identify potential interactors of SsPpe1. Our analysis showed SsRts1, encoded by the *SPSC_06610* locus, as an interacting partner of SsPpe1 (Figure 1(a)). Furthermore, GST pull-down assays confirmed the physical interaction between SsPpe1 and SsRts1 (Figure 1(b)). These results collectively support the existence of a specific interaction between SsRts1 and SsPpe1.

### *Characterization of SsRts1 in* S. scitamineum

The full-length *Ssrts1* sequence spans 2504 bp and encodes a polypeptide of 732 amino acids. To identify homologs of SsRts1, we performed a BLASTp search against the published fungal genomes. Evolutionary analysis revealed that SsRts1 is most closely related to SrRts1 (CBQ73266.1) from *Sporisorium reilianum* (Figure 1(c)). Additionally, Figure 1(c) showed that the SsRts1 homologs in *U. maydis* and *P. oryzae* have sequence identities of 93.20% and 64.02%, respectively. Protein sequence alignment demonstrated that Rts1 orthologs are highly conserved among smut fungi and all contain a B56 domain. This indicates that SsRts1 belongs to the PP2A regulatory subunit of B′ family.

### *SsRts1 is required for sporidia growth, morphogenesis, and mitosis in* S. scitamineum

To investigate the biological function of SsRts1 in *S. scitamineum*, we constructed the *Ssrts1* knockout mutant using replacement with a hygromycin resistance gene (*HPT*) in both mating types, JG36 (MAT-1) and JG35 (MAT-2). The correct deletion of the *Ssrts1* gene was confirmed by PCR analysis of purified genomic DNA from each strain (Figure S1). To ensure that the phenotypic changes were actually caused by the deletion of *Ssrts1*, the Δ*Ssrts1* mutants were complemented by re-introducing a copy of the wild-type *Ssrts1* gene. PCR analysis verified that both the *Ssrts1* gene and the *zeocin*-resistant gene had been successfully integrated into the Δ*Ssrts1* mutant genome, suggesting that the complemented strain C-Δ*Ssrts1* mutants were successfully constructed (Figure S2).

**Figure 2.**
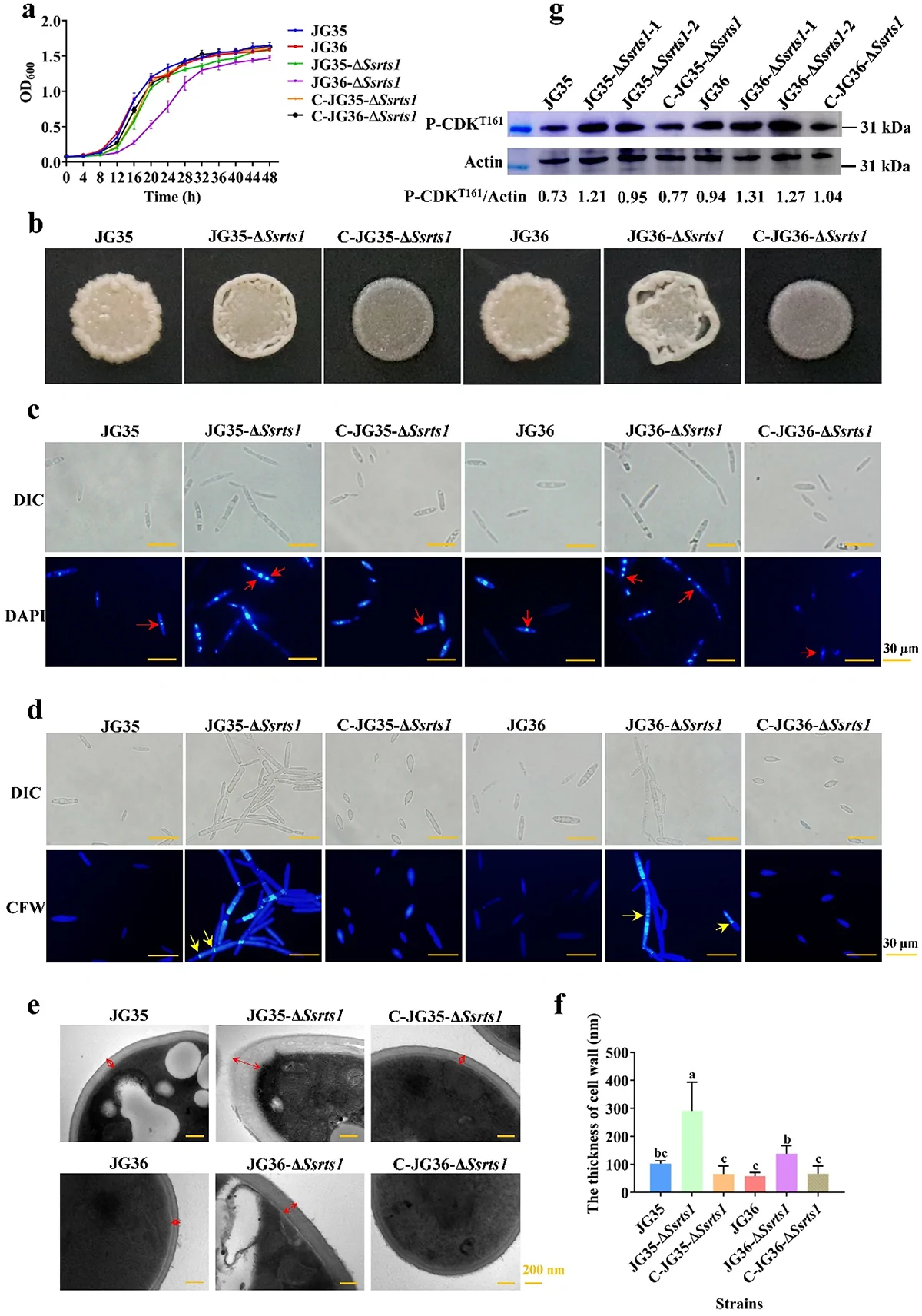
The Δ*Ssrts1* mutants exhibit defects in growth, morphology and mitosis. (a) Growth curves of *S. scitamineum*. WT and the Δ*Ssrts1* mutant strains were cultured at 28°C for 48 h, and the growth was monitored by measuring OD_600_ every 4 h. The data represent the mean ± standard error of three independent biological replicates (*n* = 3). (b) Colony morphology of the mutants on YePSA medium. Photographs were taken at 3 days after inoculation. (c) The haploid sporidia (*n* = 21) were stained by DAPI to observe the nuclear distribution. Red arrows indicate nuclei. (d) The haploid sporidia (*n* = 23) were stained by CFW to observe the chitin deposition. Yellow arrows indicate chitin deposition. (e) The cell wall thickness of WT and Δ*Ssrts1* mutant strains was observed by TEM. (f) Statistical analysis of the cell wall thickness of WT and Δ*Ssrts1* mutant strains. The data represent the mean ± standard error of three independent biological replicates (*n* = 3). Distinct letters above the bars denote significant differences in treatment (ANOVA and Tukey’s test, *p <* 0.05). (g) Comparisons of CDK1^T161^ phosphorylation among wild-type, Δ*Ssrts1* and C-Δ*Ssrts1* strains. Western blots were performed with total proteins from sporidia of wild-type, Δ*Ssrts1* and C-Δ*Ssrts1* strains. The signal corresponding to phosphorylated CDK1 was detected by binding of the antiphospho-CDK1^T161^ antibody with the Actin antibody as a control. The ImageJ software was used to calculate the grayscale values. P-CDK1^T161^1/Actin represents the ratio of the grayscale values of two bands. (a, b, e, f, g) representative images from three independent biological replicates are shown above.

To examine the potential roles of *Ssrts1* in haploid sporidia growth of *S. scitamineum*, wild-type strains, *Ssrts1* deletion mutants, and complementation strains were cultured in liquid YePS medium. The growth curve showed that the growth rate of the JG36-Δ*Ssrts1* strains was slower than that of the wild-type and complementation strains (Figure 2(a)). The morphology of sporidia in Δ*Ssrts1* mutants was further studied. When cultured on solid medium, the Δ*Ssrts1* sporidia colonies appeared wrinkled and less smooth compared to the wild-type strain (Figure 2(b)). Microscopic examination revealed the frequent presence of elongated pseudohyphae in the Δ*Ssrts1* sporidial culture, which was rarely observed in the WT (Figure 2(c, d)). Collectively, these results indicate that the SsRts1 is required for normal sporidia growth and morphogenesis in *S. scitamineum*.

Based on the elongated sporidia phenotype observed in Δ*Ssrts1* mutants, we hypothesized that this morphological alteration might result from aberrant mitosis during sporidia development. We assessed nuclear number and distribution, while WT sporidia typically contained one or two nuclei, the elongated pseudohyphal cells of Δ*Ssrts1* mutants (*n* = 21) displayed multiple nuclei (Figure 2(c)). Further visualization of chitin deposition and cell wall (Figure 2(d)) revealed that Δ*Ssrts1* mutants (*n* = 23) exhibited abnormal distribution of both cellulose and chitin compared with the wild-type, indicating potential defects in cell wall integrity. Transmission electron microscopy (TEM) analysis was therefore conducted to examine cell wall ultrastructure, revealing a significantly thicker cell wall in Δ*Ssrts1* mutants relative to WT (Figure 2(e, f)). These phenotypes in the Δ*Ssrts1* mutants may be caused by abnormal mitosis.

To investigate the role of SsRts1 in mitosis, we analyzed the expression levels of five Rho GTPase genes involved in cell wall biosynthesis and mitosis in both JG35 (WT) and JG35-Δ*Ssrts1* strains. Quantitative results indicated that all five Rho GTPase genes were significantly downregulated in the Δ*Ssrts1* mutants (Figure S3), indicating that SsRts1 plays a role in regulating the expression of Rho GTPase family genes in *S. scitamineum*. To further verify whether SsRts1 regulates mitosis, we examined the phosphorylation level of CDK1^T161^ in both WT and Δ*Ssrts1* mutant strains. CDK1 phosphorylation is known to act as a switch for the mitotic process [20]. In cyclin B1-CDK1 complexes, the activating phosphorylation at T161 and the inhibitory phosphorylation at T14 coordinately regulate cell cycle progression. However, excessive T161 phosphorylation leads to sustained CDK1 activation, thereby resulting in catastrophic mitosis [21]. As shown in Figure 2(g), the Δ*Ssrts1* mutants exhibited a significantly increased level of CDK1^T161^ phosphorylation compared with the WT. Overall, these results indicate that SsRts1 participates in the regulation of mitosis process in *S. scitamineum*.

### *SsRts1 is involved in cell wall stress, oxidative and high osmotic stress response in* S. scitamineum

**Figure 3.**
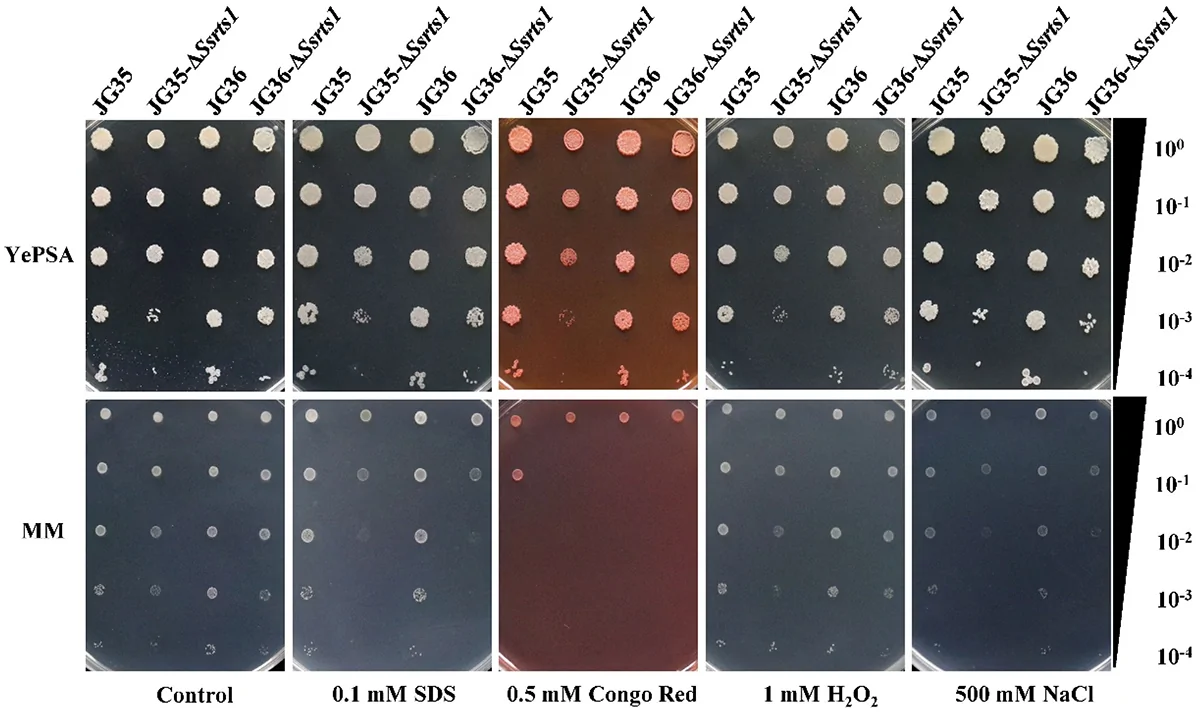
Assessment of the tolerance of Δ*Ssrts1* strains toward stressful conditions. The wild-type (JG35 and JG36) and deletion mutant strains (JG35-Δ*Ssrts1* and JG36-Δ*Ssrts1*) were spotted onto YePSA or MM medium supplemented with the indicated stress agents and incubated at 28°C for 3 days. Representative images from three independent biological replicates were shown.

To investigate the role of *Ssrts1* in the adaptation to infection-related stresses, we compared the radial growth of WT and *Ssrts1* deletion mutant strains on YePSA and MM media supplemented with the following stressors: sodium dodecyl sulfate (SDS, a membrane-disrupting agent), Congo red (CR, a cell wall-disturbing agent), hydrogen peroxide (H_2_O_2_, an oxidative stress agent), and NaCl (an osmotic stress agent). As shown in Figure 3, the Δ*Ssrts1* mutants exhibited significantly increased sensitivity to cell wall integrity (CWI) stress, oxidative stress and high osmotic stress induced by SDS, CR, H_2_O_2_ and NaCl, compared to the WT strains.

Mps1 is a downstream mitogen-activated protein kinase (MAPK) in the CWI pathway and plays an important role in maintaining fungal cell wall integrity [22]. To explore whether SsRts1 participates in cell wall stress response and modulates the CWI signaling pathway, we further examined the phosphorylation level of Mps1 in WT, Δ*Ssrts1* and complement strains. As shown in Figure S4, the phosphorylation level of Mps1 was increased significantly in Δ*Ssrts1* mutant strains compared to wild-type strains. These findings suggest that SsRts1 participates in responses to oxidative and high osmotic stresses, and negatively modulates the phosphorylation status of the CWI-MAPK cascade to sustain cell wall integrity in *S. scitamineum*.

### *Deletion of* Ssrts1 *results in the accumulation of autophagosome in* S. scitamineum

**Figure 4.**
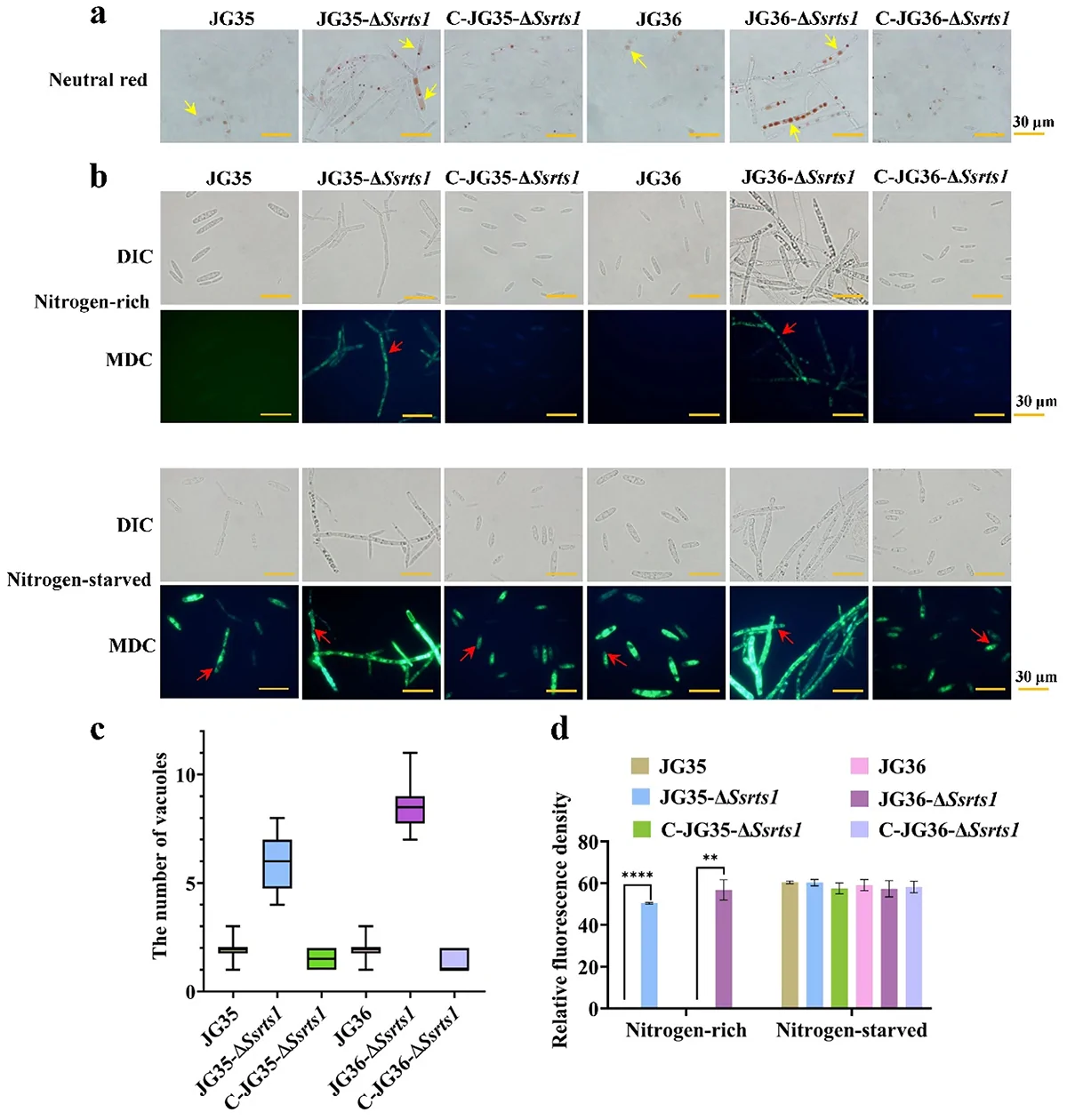
SsRts1 negatively regulates the autophagy activation in *S. scitamineum*. (a) The haploid sporidia stained with neutral red to observe the vacuoles distribution. Yellow arrows indicate vacuoles. (b) The haploid sporidia stained with MDC to observe the autophagosome distribution. Red arrows indicate autophagosome. (c) Statistical analysis of the number of vacuoles in wild-type, Δ*Ssrts1* and complemented strains. The data represent the mean ± standard error of ten biological replicates (*n* = 10). (d) Quantification of fluorescence intensity shown in (b). Samples from different strains were measured using ImageJ software. The data represent the mean ± standard error of three biological replicates (*n* = 3), **p < 0.01, ****p < 0.0001 (Student’s *t*-test). Images were shown as above.

Previous studies established that the elongated sporidia phenotype associated with autophagy. Then we first stained sporidia of Δ*Ssrts1* mutants with neutral red to examine vacuolar distribution. As shown in Figure 4(a,c), the Δ*Ssrts1* mutants exhibited a significant increase in both the number and size of vacuoles compared to the WT and C-Δ*Ssrts1* strains.

We therefore suspected that the larger vacuoles may be associated with the accumulation of autophagosome. To further evaluate the role of *Ssrts1* in autophagy, we cultured the sporidia under nitrogen-rich or nitrogen-starved conditions, then stained the sporidia with the autophagosome-specific dye monodansylcadaverine (MDC). Under nitrogen-rich conditions, no autophagosome was observed in wild-type or complemented sporidia, whereas numerous autophagosomes appeared in the vacuole lumen of both JG35-Δ*Ssrts1* and JG36-Δ*Ssrts1* (Figure 4(b, d)). Following nitrogen starvation, autophagic vacuoles were found to accumulate in the wild-type, complemented and Δ*Ssrts1* strains (Figure 4(b)). These results suggest that the SsRts1 involved the autophagy process in *S. scitamineum*.

### *SsRts1 is essential for sexual mating and filamentation of* S. scitamineum

**Figure 5.**
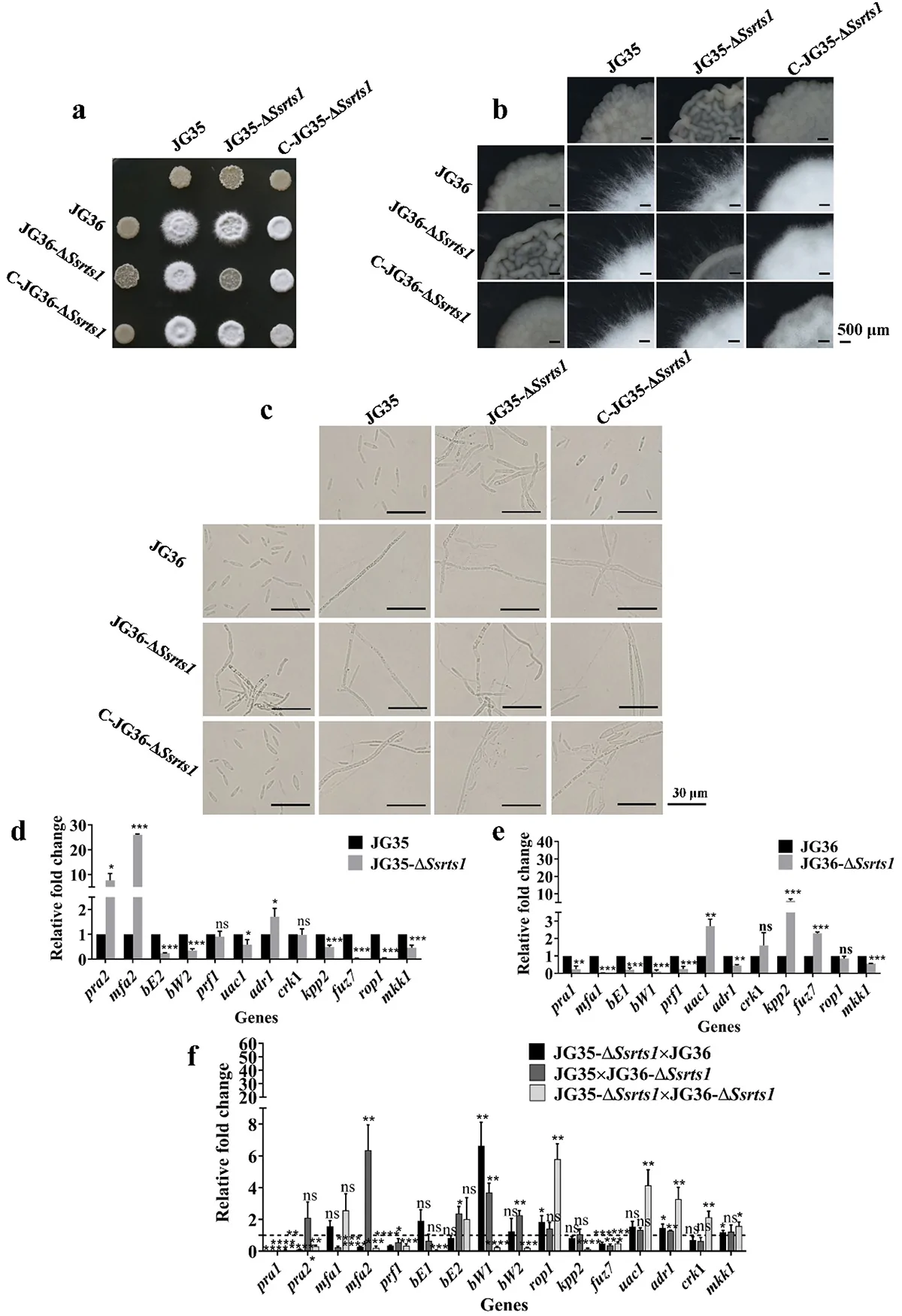
*Ssrts1* is essential for *S. scitamineum* mating and filamentation. (a) Wild-type strains and *Ssrts1* deletion mutants were co-spotted on YePSA plates and incubated at 28°C for 3 days. The appearance of white, fluffy dikaryotic hyphae indicated a successful mating reaction. (b) Enlarged view of colony edges of (a). Colonies were photographed under a stereomicroscope. Bar = 500 μm. (c) Microscopic observation of (a). Bar = 30 μm. (d) RT-qPCR analysis of the relative expression of genes essential for mating and filamentous growth in haploid and dikaryon stages in JG35 and JG35-Δ*Ssrts1*. (e) JG36 and JG36-Δ*Ssrts1*. (f) Mating pairs. The dotted line indicates that the gene expression levels in the wild-type strain pairs were set to 1.0. The data represent the mean ± standard error of three independent biological replicates (*n* = 3). *p < 0.05, **p < 0.01, ***p < 0.001, ****p < 0.0001, ‘ns’ indicates no significant difference (Student’s *t*-test).

To investigate the role of *Ssrts1* in sexual mating and filamentous growth, haploid cells of opposite mating types were co-cultured on YePSA medium. After three days, the wild-type strains JG35×JG36 formed white, fluffy colonies with typical dikaryotic filamentous hyphae. In contrast, the JG35-Δ*Ssrts1*×JG36-Δ*Ssrts1* combination failed to produce fluffy colonies or filamentous growth, indicating that deletion of *Ssrts1* impaired the mating and filamentation capabilities of the fungal strains. However, the complemented strains C-JG35-Δ*Ssrts1*×C-JG36-Δ*Ssrts1* restored the fluffy white colony and exhibited normal dikaryotic hyphae, which were indistinguishable from WT (Figure 5(a-c) and Figure S5). Furthermore, both the JG35-Δ*Ssrts1*×JG36 and JG35×JG36-Δ*Ssrts1* combinations exhibited a colony phenotype similar to the wild type (Figure 5(b)), suggesting that mating and filamentation are attenuated only when both *Ssrts1* alleles are deleted, in an allele-dosage-dependent manner.

To further investigate the relationship between *Ssrts1* and the pheromone signaling pathway, we analyzed the expression levels of genes involved in mating and filamentation using RT-qPCR. In the JG35-Δ*Ssrts1* mutant, several key genes were downregulated, including *bE2* and *bW2*, which encode a heterodimeric transcription factor, along with *uac1*, *kpp2*, *fuz7*, *rop1* and *mkk1*, all key components of the MAPK signaling pathway. By contrast, the mating factor gene *mfa2*, the pheromone receptor gene *pra2* and *adr1* were upregulated (Figure 5(d)). In JG36-Δ*Ssrts1*, however, *pra1*, *mfa1*, *bE1*, *bW1*, *adr1*, *mkk1* and the pheromone response factor gene *prf1* were downregulated, while *uac1*, *kpp2* and *fuz7* genes showed increased expression relative to wild-type controls (Figure 5(e)). We further examined the expression patterns of these genes when one or both *Ssrts1* alleles were deleted. In JG35-Δ*Ssrts1*×JG36, the expression of *pra1*, *pra2*, *mfa2*, *prf1* and *fuz7* were significantly downregulated, whereas *bW1*, *rop1*, *adr1* and *mkk1* were significantly upregulated. In JG35×JG36-Δ*Ssrts1*, *pra1*, *mfa1*, *prf1* and *fuz7* were significantly downregulated, while *mfa2*, *bE2*, *bW1*, *bW2* and *adr1* were significantly upregulated. When both *Ssrts1* alleles were deleted, a-locus genes (*pra1/2*, *mfa2*), b-locus genes (*bE1*, *bW1/2*), and the pheromone response factor gene *prf1* were downregulated. Among MAPK signaling pathway genes, *rop1, uac1*, *adr1*, *crk1*, and *mkk1* were upregulated, whereas *kpp2* and *fuz7* were not (Figure 5(f)). Collectively, these results suggest that altered expression patterns of pheromone and MAPK pathway genes likely contribute to the mating deficiency in *Ssrts1* deletion mutants.

### *SsRts1 is crucial for the full virulence in* S. scitamineum

**Figure 6.**
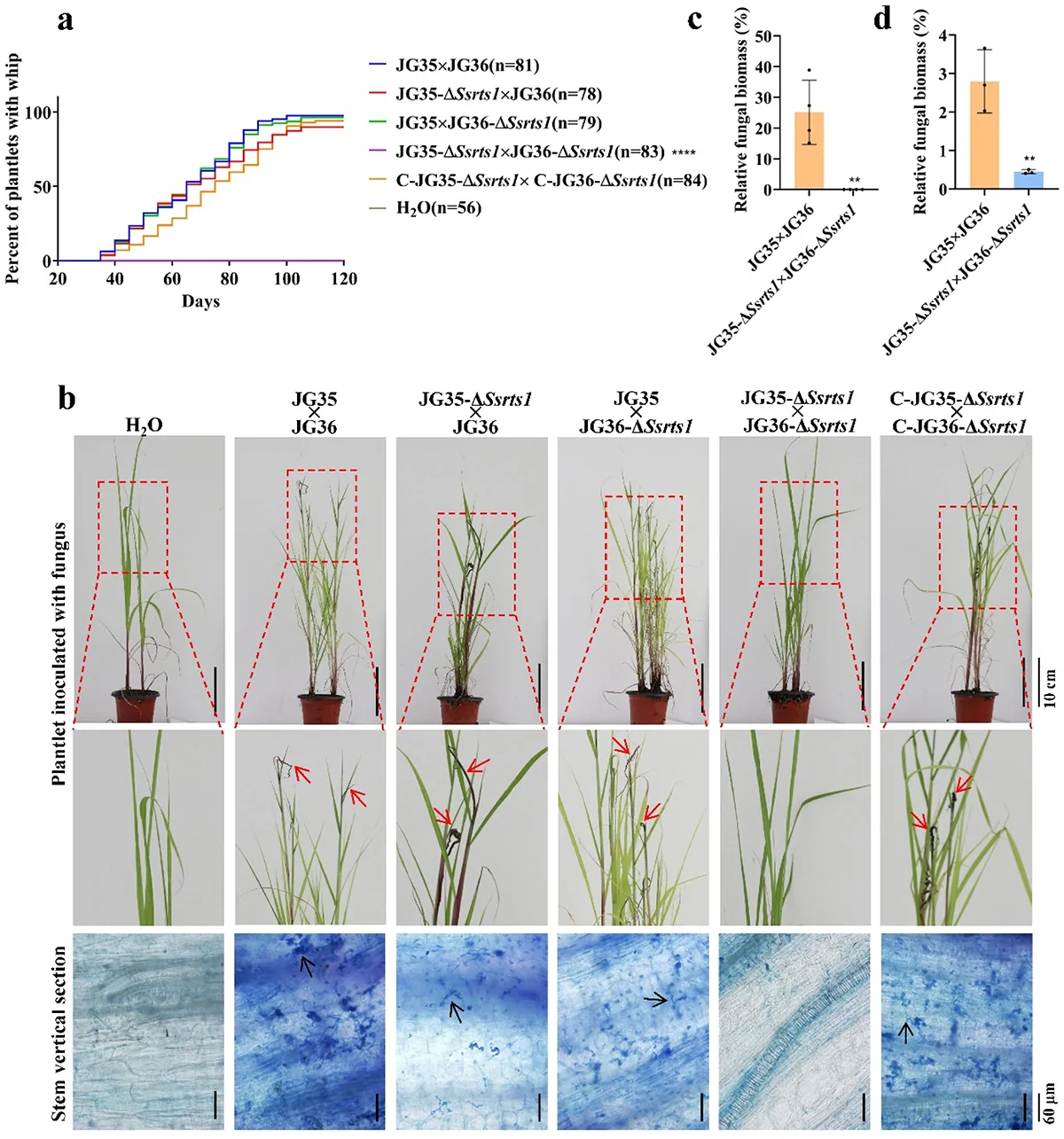
*Ssrts1* required for full pathogenicity of *S. scitamineum*. (a) Progression of whip development induced by WT strains, *Ssrts1* deletion mutants and complementation strains. Three independent biological repeats were performed, ****p < 0.0001 (Student’s *t*-test). (b) Microscopic sections of sugarcane cells infected by WT strains, *Ssrts1* deletion mutants and complementation strains. Red arrows indicate whips. Black arrows indicate fungal hyphae. (c) Relative fungal biomass in sugarcane inoculated by root-dip inoculation. The data represent the mean ± standard error of four independent biological replicates (*n* = 4), **p < 0.01 (Student’s *t*-test). (d) Relative fungal biomass in sugarcane inoculated by bud-injection inoculation. The data represent the mean ± standard error of three independent biological replicates (*n* = 3), **p < 0.01 (Student’s *t*-test).

To investigate the impact of *Ssrts1* deletion on virulence, we conducted virulence assays using tissue culture seedlings derived from the smut-susceptible sugarcane variety ROC22. At 120 days post-inoculation, 97.5% of sugarcane inoculated with the wild-type strains (JG35×JG36) displayed “black whip” symptoms, 89.7% and 96.2% of whips developed in plants inoculated with JG35-Δ*Ssrts1*×JG36 and JG35×JG36-Δ*Ssrts1*, respectively. In contrast, none of the seedlings infected with JG35-Δ*Ssrts1*×JG36-Δ*Ssrts1* developed whip symptoms. The complemented strains C- JG35-Δ*Ssrts1*×C-JG36-Δ*Ssrts1* restored the whip formation rate to 94.0% (Figure 6(a) and Table 1). The results show that the deletion of both alleles of *Ssrts1* gene led to a complete loss of virulence in *S. scitamineum*.

**Table 1.** Incidence of sugarcane plantlets inoculated with Δ*Ssrts1* mutants.

Sample	Total Plantlets	Black Whip	Incidence rate (%)	Infection rate (%)
JG35×JG36	81	79	97.5	98.8
JG35×JG36-Δ*Ssrts1*	79	76	96.2	97.5
JG35-Δ*Ssrts1*×JG36	78	70	89.7	94.9
JG35-Δ*Ssrts1*×JG36-Δ*Ssrts1*	83	0	0	0
C-JG35-Δ*Ssrts1*×C-JG36-Δ*Ssrts1*	84	79	94.0	96.4
H_2_O	56	0	0	0

We further analyzed the infection rate of the wild type, Δ*Ssrts1* and the complemented strains. Previous reports showed that sugarcane is considered infected when fungal hyphae are present [23]. Microscopic examination of tissues revealed that obvious fungal hyphae were observed in the meristematic tissues of sugarcane inoculated with the wild-type strain, whereas no fungal hyphae were detected in those inoculated with JG35-Δ*Ssrts1* × JG36-Δ*Ssrts1* (Figure 6(b), Table 1). To further analyze fungal biomass in inoculated plantlets that did not exhibit black whip symptoms, we quantified *S. scitamineum* colonization. Abundant fungal signals were detected in plantlets inoculated with WT strains, whereas no fungi were found in those inoculated with JG35-Δ*Ssrts1* × JG36-Δ*Ssrts1* (Figure 6(c)), indicating that the mutants were blocked at the penetration stage.

Additionally, given that dikaryotic hyphae invade sugarcane bud tissues under natural field conditions [24], we used needle puncture to inoculate buds and assessed the infection capability and fungal biomass of the JG35-Δ*Ssrts1* × JG36-Δ*Ssrts1* mutant. The results revealed that the fungal biomass in buds inoculated with the mutant was significantly lower than that in buds inoculated with WT strains (Figure 6(d)), further indicating that the mutant is impaired at the penetration stage. In summary, these results demonstrate that deletion of both *Ssrts1* alleles significantly reduced the virulence and infection rate of *S. scitamineum.*

### SsRts1 regulates key biological processes at the transcriptomic level

To better understand the regulation mechanism of *Ssrts1* in *S. scitamineum*, we compared the transcriptomes of Δ*Ssrts1* and wild-type strains using high-throughput sequencing. The JG35, JG35-Δ*Ssrts1*, JG36, and JG36-Δ*Ssrts1* strains yielded 39.4–45.6 million, 42.3–46.5 million, 39.8–47.3 million, and 42.7–46.9 million reads, respectively. After removing adapter sequences and low-quality reads, 38.2–44.6 million, 41.4–45.2 million, 38.9–46.1 million and 41.8–46.0 million reads remained for subsequent analysis (Table 2).

**Table 2.** Summary of sequencing date and read-alignment statistics from RNA-Seq.

Samples	Raw date	Clean date	Unique mapped reads
JG35-1	44,519,346	43,777,452	41,537,724 (94.88%)
JG35-2	45,554,420	44,572,414	42,410,250 (95.15%)
JG35-3	39,383,296	38,199,300	36,060,912 (94.40%)
JG35-Δ*Ssrts1*-1	46,479,612	45,150,802	40,684,192 (90.11%)
JG35-Δ*Ssrts1*-2	42,325,362	41,389,894	40,083,991 (96.84%)
JG35-Δ*Ssrts1*-3	44,078,250	43,162,006	41,499,0 72 (96.15%)
JG36-1	39,843,926	38,854,892	36,876,513 (94.91%)
JG36-2	41,785,124	40,787,938	38,838,971 (95.22%)
JG36-3	46,274,352	46,147,300	44,574,362 (96.59%)
JG36-Δ*Ssrts1*-1	46,949,674	46,035,636	43,812,310 (95.17%)
JG36-Δ*Ssrts1*-2	42,732,664	41,777,128	39,464,985 (94.47%)
JG36-Δ*Ssrts1*-3	44,176,576	43,539,192	40,110,514 (92.13%)

**Figure 7.**
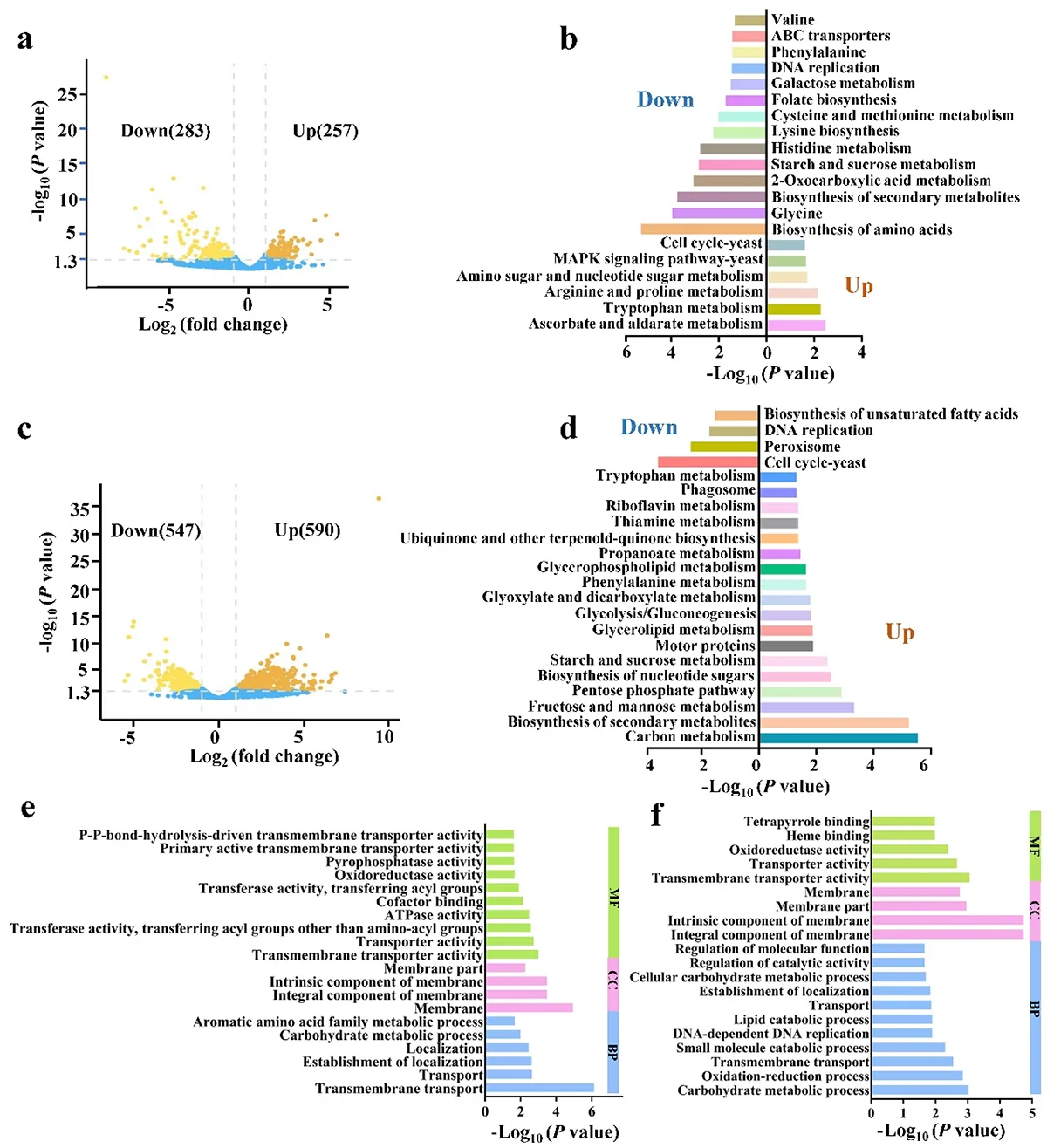
RNA-seq date of the wild-type and Δ*Ssrts1* strains. (a) The DEGs between the JG35 and JG35-Δ*Ssrts1* strains were shown with a volcano plot. mRNAs log_2_FC > 1 in JG35-Δ*Ssrts1* relative to JG35 (*p* value < 0.05) were highlighted in orange. mRNAs log_2_FC < −1 in JG35-Δ*Ssrts1* relative to JG35 (*p* value < 0.05) were highlighted in yellow. DEGs, differentially expressed genes. (b) KEGG pathway enrichment analysis of DEGs between JG35 and JG35-Δ*Ssrts1* strains. (c) The DEGs between the JG36 and JG36-Δ*Ssrts1* strains were shown with a volcano plot. mRNAs log_2_FC > 1 in JG36-Δ*Ssrts1* relative to JG36 (*p* value < 0.05) were highlighted in orange. mRNAs log_2_FC < −1 in JG36-Δ*Ssrts1* relative to JG36 (*p* value <0.05) were highlighted in yellow. (d) KEGG pathway enrichment analysis of DEGs between JG36 and JG36-Δ*Ssrts1* strains. (e) GO-based enrichment analysis of DEGs between JG35 and JG35-Δ*Ssrts1* strains. (f) GO-based enrichment analysis of DEGs between JG36 and JG36-Δ*Ssrts1* strains.

RNA-seq analysis identified 540 differentially expressed genes (DEGs) in JG35-Δ*Ssrts1* compared to JG35, comprising 283 down-regulated genes and 257 up-regulated genes [*p* < 0.05 and |log_2_ fold change (FC)| ≥1] (Figure 7(a)). The reliability of the RNA-seq data was assessed by RT-qPCR analysis of 10 randomly selected DEGs. RT-qPCR confirmed that the expression trends of the 10 DEGs were consistent with RNA-seq (Figure S6a). The 540 DEGs were then analyzed by Kyoto Encyclopedia of Genes and Genomes (KEGG) pathway and gene ontology (GO) enrichment analysis. In detail, the DEGs downregulated in JG35-Δ*Ssrts1* were significantly enriched in biosynthesis of amino acids, glycine and biosynthesis of secondary metabolites; the upregulated genes were mostly involved in ascorbate and aldarate metabolism, tryptophan metabolism, arginine and proline metabolism (Figure 7(b)). Furthermore, these DEGs were used to perform Gene Ontology (GO) enrichment analysis, the results showed that the DEGs were mostly involved in transmembrane transport, membrane and transmembrane transporter activity (Figure 7(e)). For JG36-Δ*Ssrts1* compared with JG36, 1137 DEGs were identified, including 547 down-regulated genes and 590 up-regulated genes (Figure 7(c)). The RT-qPCR results were consistent with the RNA-seq data, confirming the reliability of the transcriptome data (Figure S6b). KEGG enrichment analysis revealed that downregulated genes were significantly enriched in cell cycle, peroxisome and DNA replication; the upregulated genes were mostly involved in carbon metabolism, biosynthesis of secondary metabolites and fructose and mannose metabolism (Figure 7(d)). GO enrichment analysis revealed that DEGs were significantly enriched in intrinsic component of membrane, transmembrane transporter activity and carbohydrate metabolic process (Figure 7(f)).

**Figure 8.**
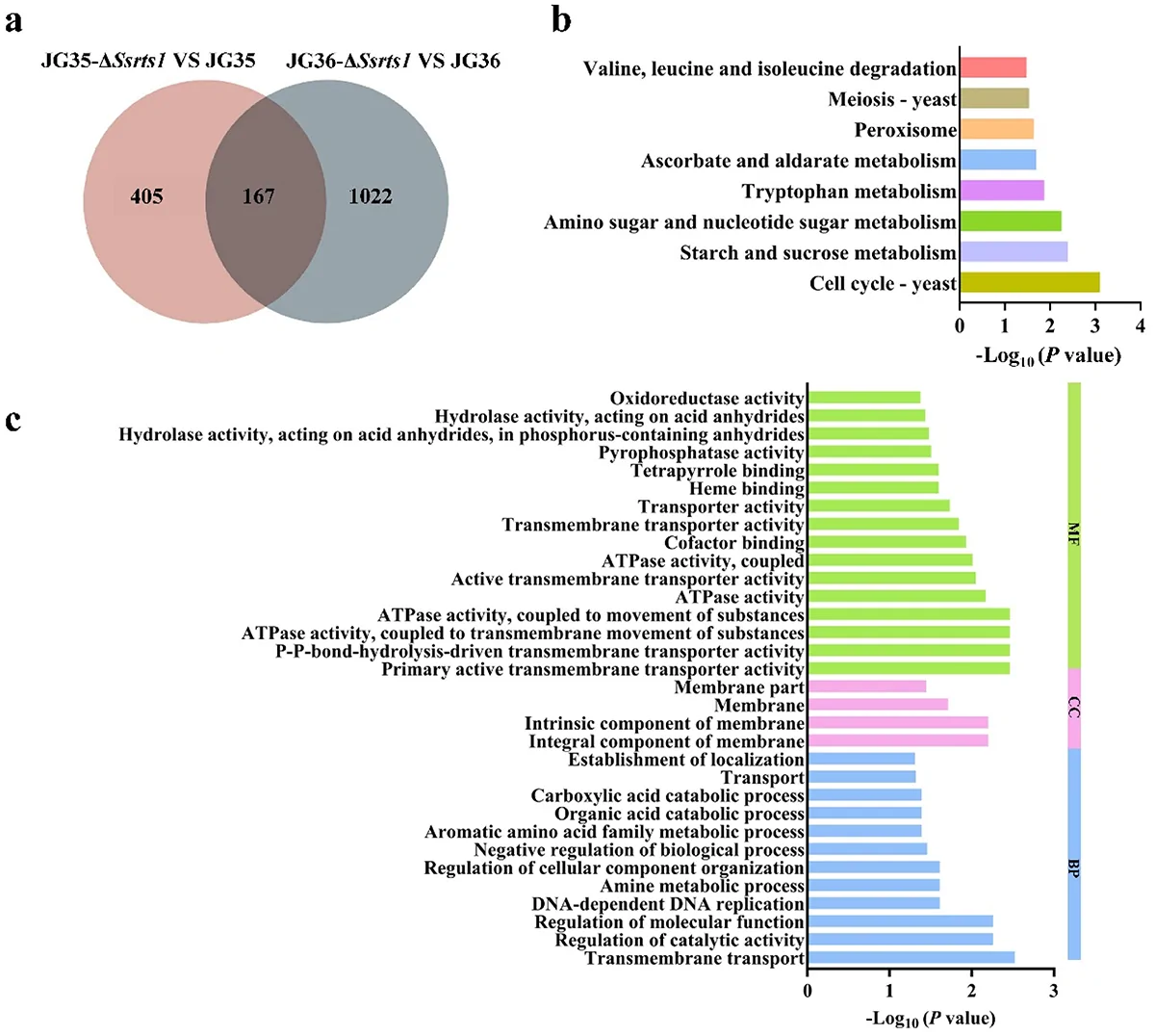
Analysis of the common DEGs between the JG35-ΔSsrts1 vs JG35 and JG36-ΔSsrts1 vs JG36 comparisons. (a) The Venn diagram of the common DEGs in the JG35-Δ*Ssrts1* and JG36-Δ*Ssrts1* groups. (b) KEGG pathway enrichment analysis of the common DEGs. (c) GO-based enrichment analysis of the common DEGs in terms of biological process (BP), cellular component (CC), and molecular function (MF).

Since both JG35-Δ*Ssrts1* and JG36-Δ*Ssrts1* mutants exhibited similar sporidia phenotype, we further analyzed the 167 common DEGs between the JG35-Δ*Ssrts1* vs JG35 and JG36-Δ*Ssrts1* vs JG36 comparisons using a Venn diagram (Figure 8(a)). The 167 DEGs were then analyzed by Kyoto Encyclopedia of Genes and Genomes (KEGG) pathway and gene ontology (GO) enrichment analysis. As shown in Figure 8(b,c), the common DEGs were significantly enriched in KEGG pathways including cell cycle and tryptophan metabolism, and in GO terms such as ATPase activity and transmembrane transport. The results indicate that SsRts1 could be involved in ATPase activity, cell cycle, transmembrane transport and tryptophan metabolism.

### SsRts1 is involved in tryptophan metabolism pathway

**Figure 9.**
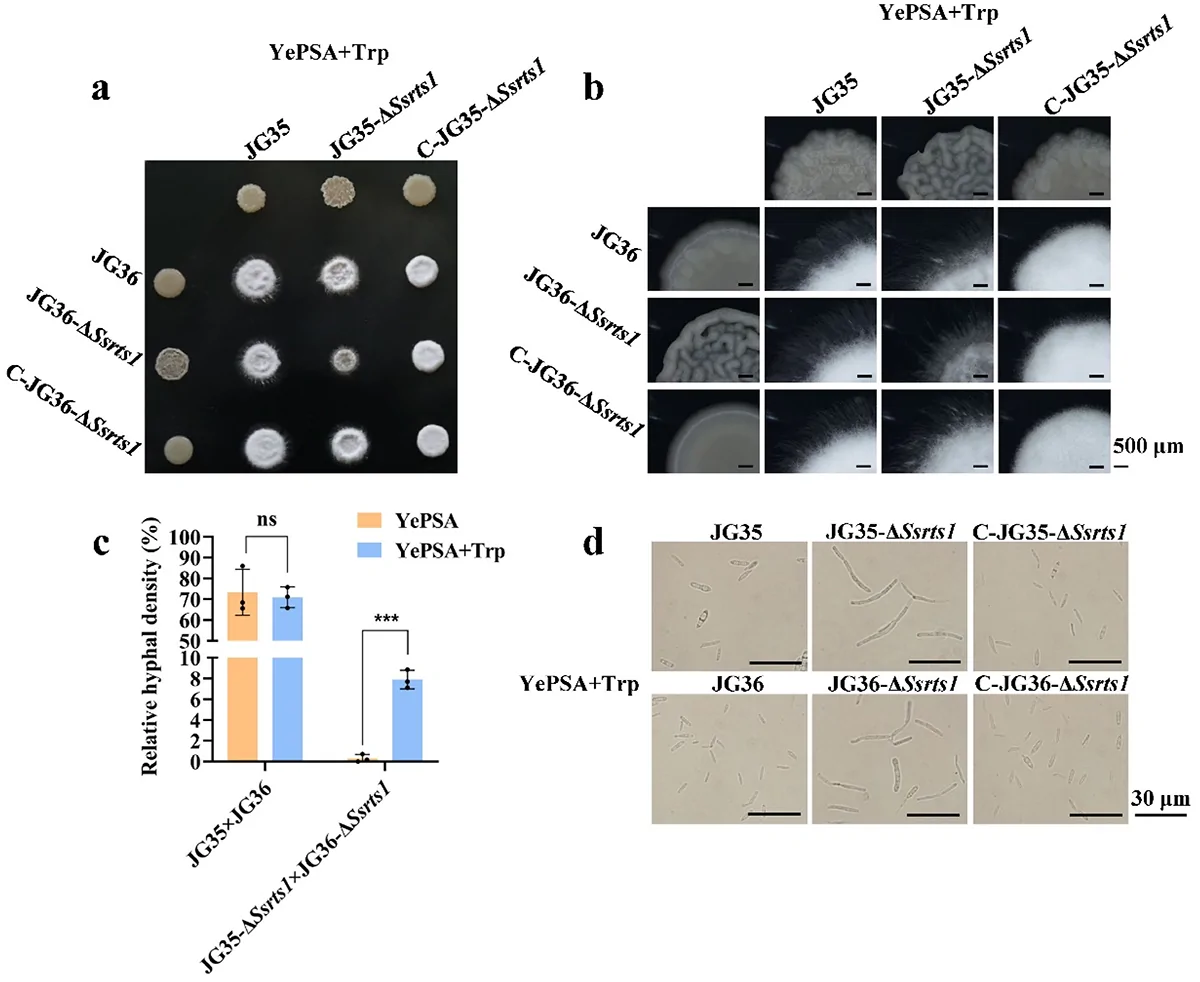
SsRts1 is involved in tryptophan metabolism. (a) Representative images show that exogenous supplementation of Trp (200 µM) can partially restore the filamentous growth defect of the Δ*Ssrts1* mutants. (b) Images are enlarged colony edges showing the fluffy hyphae. Colonies were photographed under a stereomicroscope. Bar = 500 μm. (c) Microscopic observation of (a). Bar = 30 μm. (d) Quantification of hyphal intensity shown in (a). Colonies of wild-type and JG35-Δ*Ssrts1*×JG36-Δ*Ssrts1* were measured using ImageJ software. The data represent the mean ± standard error of three biological replicates (*n* = 3), ***p < 0.001 (Student’s *t*-test). Images were shown as above.

The above transcriptome analysis revealed significant alterations in the expression levels of tryptophan (Trp) metabolism genes in Δ*Ssrts1* mutants (Figure 8(b)). To examine the relationship of SsRts1 and tryptophan metabolism, we supplemented different concentrations of Trp to the mating culture and assessed filamentous growth in both JG35 × JG36 and JG35-Δ*Ssrts1* × JG36-Δ*Ssrts1*. The results showed that exogenous supplementation of Trp at low concentrations (50, 100 and 150 µM) failed to restore filamentous growth (Figure S7). However, exogenous supplementation of Trp (200 µM) could significantly promote filamentous growth of JG35-Δ*Ssrts1*×JG36-Δ*Ssrts1* (Figure 9(a–c)), but had no effect on sporidia morphology of JG35-Δ*Ssrts1* and JG36-Δ*Ssrts1* strains (Figure 9(d)). These findings indicate that SsRts1 likely regulates mating/filamentation in *S. scitamineum* through the tryptophan metabolism pathway.

## Discussion

Reversible phosphorylation of proteins acts like a molecular switch that regulates many biological processes in fungi. In recent years, many protein kinases have been demonstrated to be involved in regulating the sexual mating/filamentation and virulence in *S. scitamineum* (Cai et al., 2021a [9,12]). However, fewer studies have focused on the roles of protein phosphatases. As a key regulator of phosphorylation, protein phosphatase plays an essential role in maintaining the phosphorylation levels of substrate proteins and ensuring normal signal transduction. PP2A is a widely conserved Ser/Thr phosphatases, and functions as a trimeric protein complex that composed of a catalytic subunit, a scaffold subunit and one alternative regulator subunit [25]. The PP2A regulator subunits consist of four members B (B55), B′ (B56), B′′ (B72) and B′′′ (Striatin), each of which belongs to different families. The regulator subunits determine the substrate specificity, subcellular localization and catalytic activity of the PP2A holoenzymes [26]. In the recent study, we identified the PP2A-like phosphatase SsPpe1 in *S. scitamineum* and characterized its essential roles of mating/filamentation and virulence [19]. This study presents evidence that SsRts1, serving as a regulatory subunit, interacts with SsPpe1 (Figure 1).

Loss of *Ssrts1* caused slower sporidia growth, abnormal morphology. Interestingly, the Δ*Ssrts1* mutants have a pseudohyphal morphology consisting of chains of slightly elongated cells under yeast growth conditions (Figure 2(c, d)). The Δ*Ssrts1* sporidia morphology is similar to *Saccharomyces cerevisiae* pseudohyphae cells. The term pseudohyphae in *S. cerevisiae* refers to elongated cells that remain connected after cell division and septum formation [27]. In *C. albicans*, deletion of the *rts1* gene caused round and enlarged cells, and seemed to undergo incomplete cell separation, producing multinucleated cells [28]. Previous studies have showed that Rts1 is required for the proportional relationship between cell size and growth rate during mitosis [29]. The pseudohyphal growth mode encompasses changes in cell cycle progression. During the telophase stage of mitosis, the nuclear membrane and nucleolus reassemble to form two daughter nuclei [30]. Meanwhile, actomyosin ring contracts, starting chitin accumulation that results in septum formation [31]. Finally, cytokinesis results in the complete separation of mother and daughter cells. We speculated that the elongated sporidia morphology in Δ*Ssrts1* mutants was caused by the cytokinesis defect. This study showed that elongated sporidia of Δ*Ssrts1* mutants have more than two nuclei (Figure 2(c)). Compared with wild-type, the chitin is mainly distributed in the middle of Δ*Ssrts1* mutants sporidia (Figure 2(d)). The process of cytokinesis involves cell wall reorganization. The cell walls of Δ*Ssrts1* mutants are significantly thicker than wild-type strains (Figure 2(e, f)). In addition, deletion of *Ssrts1* resulted in a slower growth rate and significantly increased the phosphorylation levels of the cell cycle kinase CDK1^T161^ (Figure 2(a, g)). Increased phosphorylation of CDK1^T161^ leads to its excessive activation, causing cells to skip the G2/M checkpoint and enter continuous mitosis, but unable to complete cytokinesis [21,32,33]. This is most likely the reason why Δ*Ssrts1* mutants showed pseudohyphal morphology. Rho family GTPases are known to regulate diverse physiological processes, including morphogenesis, polarity establishment, cell integrity maintenance, cytokinesis, and cellular differentiation. In yeast, the Rho GTPases are implicated in cell polarity processes and cell wall biosynthesis [34]. Consistently, the transcriptional levels of several cellular integrity, morphogenesis and cytokinesis-related Rho GTPase genes, including *SsRho1*, *SsRho2*, *SsRho3*, *SsRac1* and *SsCdc42*, were significantly decreased in the Δ*Ssrts1* mutants (Figure S3). Consistent with these studies, RNA-Seq results also showed that DEGs in the Δ*Ssrts1* mutants were significantly enriched in the cell cycle pathway (Figure 8(b)). These results all indicate that SsRts1 play an important role in regulating the mitosis of *S. scitamineum*.

Previous studies reported that deletion of the *Rts1* gene results in greater sensitivity to cell wall stress in *C. albicans* and *M. oryzae* [28,35]. In the current study, we found that deletion of *Ssrts1* lead to increased sensitivity to cell wall stress, oxidative stress and high osmolarity stress (Figure 3). In *Saccharomyces cerevisiae*, PP2A^Rts1^ via a SLiM B56-family interaction motif directly regulate MAPKAP kinase Rck2 to response high osmotic stress [36]. In *Setosphaeria turcica*, MAPKK gene *StPBS2* silence transformants displayed thicker cell wall and more sensitive to cell wall and hypertonic stress [37]. In *Candida glabrata*, deletion of the serine/threonine kinase *Elm1* gene result in elongated morphology and a thicker cell wall, as well as increased sensitively to cell-wall-damaging agent [38]. These results implied that SsRts1 may regulate the cell wall integrity pathway, the high osmolarity pathway and oxidative stress by modulating a protein kinase associated with the MAPK pathway. This study demonstrates that SsRts1 can dephosphorylate the CWI pathway kinase Mps1 to maintain the cell wall integrity (Figure S4).

A previous study demonstrated that loss of the MAPKK and MAPK gene *Sshog1* and *SsPbs2* led to elongated pseudohyphae and larger vacuoles in *S. scitamineum* [39]. In this study, the results indicated that both the volume and number of vacuoles are increased in the Δ*Ssrts1* mutants (Figure 4(a)). Old yeast cells developed thicker and robust cell walls, giant vacuoles, and increased multivesicular body-like structures [40]. In filamentous fungi, autophagosomes are closely associated with vacuolar. Autophagy induction can lead to the fusion of the autophagosomal outer membrane with the vacuolar membrane. Subsequently, autophagosome and their cargoes are degraded by hydrolytic [41]. A recent study showed that the regulatory subunit 17 (PPP1R17) can inhibit the phosphatase activities of PP1 and PP2A, thereby regulating the mitophagy [42]. To verify the relationship between SsRts1 and autophagy, we further examined the number of autophagosomes in the Δ*Ssrts1* mutants by MDC staining. The results demonstrated that the nitrogen deletion can trigger autophagy in *S. scitamineum* sporidia, as evidenced by the accumulation of autophagosome in wild-type strains. Interestingly, even under the nitrogen‑abundant condition, the Δ*Ssrts1* mutants still have a large number of autophagosome (Figure 4(b, d)). These results indicated that SsRts1 is involved in regulating the autophagy process in the *S. scitamineum*. Studies have shown that PP2A is prominent in regulating physiological processes, possibly related to autophagy-related protein phosphorylation. PP2A is important in the autophagy induction of *C. albicans* by participating in Atg13 phosphorylation, followed by Atg1 activation [43]. PP2A-Cdc55 and PP2A-Rts1, which are activated by inactivation of TORC1, are required for sufficient Atg13 dephosphorylation and autophagy induction after TORC1 inactivation in budding yeast [44]. The PP2A-like protein Ppg1 and its associated Far complex cooperatively inhibit mitophagy by counteracting the phosphorylation of the mitophagy receptor Atg32 by casein kinase 2 [45]. Overall, these results support that the SsRts1 may regulate autophagy process by modulating autophagy-related protein in *S. scitamineum*.

*S*. *scitamineum* as a typical dimorphic fungus, sexual mating/filamentation is important for the pathogenicity. Amino acid metabolic pathways are involved in regulating pathogenicity [46]. In the present work, we demonstrated that deletion of *Ssrts1* results in the sexual mating/filamentation defect in the *S. scitamineum* (Figure 5(a–c)). The defects may be caused by changes in the expression levels of the sexual mating and MAPK-related genes in the Δ*Ssrts1* mutants (Figure 5(d–f)). Furthermore, the transcriptome analysis indicates that SsRts1 regulates tryptophan metabolism pathway (Figure 8(b)). Recent studies have demonstrated that tryptophan metabolism is crucial for fungal growth, development and the production of many downstream metabolites [47,48]. It has been reported that aminotransferase SsAro8 was essential for mating /filamentation in *S. scitamineum* [49]. Additionally, exogenous addition of tryptophan could restore mating/filamentation defect in the Δ*Ssaro8* mutants, indicating that products of tryptophan metabolism may be involved in *S. scitamineum* mating/filamentation regulation [49]. Our study showed that exogenous addition of tryptophan also could restore mating/ filamentation defect in the Δ*Ssrts1* mutants, further indicating that SsRts1 is involved in the tryptophan metabolism pathway (Figure 9).

In *Magnaporthe oryzae*, deletion of the B56 regulatory subunit leads to severe defects in vegetative hyphal growth, conidia formation, cell wall integrity and pathogenicity [35]. The two PP2A regulatory subunits Ppr1 and Ppr2, which are homologous of *S. cerevisiae* proteins Cdc55 and Rts1, play distinct roles in regulating fumonisin biosynthesis and fungal development in the maize pathogen *Fusarium verticillioides* [50]. The regulatory subunit SsRts1 also plays a crucial role in *S. scitamineum*. Compared to the JG35×JG36 strains, the virulence of the JG35-Δ*Ssrts1*×JG36 and JG35×JG36-Δ*Ssrts1* did not decrease significantly, but the JG35-Δ*Ssrts1*×JG36-Δ*Ssrts1* strains completely lost its pathogenicity (Figure 6(a)). This result is consistent with Δ*Ssppe1* strains [19]. In addition, the sugarcane plantlets were inoculated with the JG35-Δ*Ssrts1*×JG36-Δ*Ssrts1* strains, no fungal hyphae were observed (Figure 6(b, c)). These results indicate that *Ssrts1* is a qualitative factor for infection, only lacking both alleles would drastically reduce the infection rate.

Based on the comparative transcriptome analysis of JG35-Δ*Ssrts1* vs JG35 and JG36-Δ*Ssrts1* vs JG36, we found that cell cycle related genes and ATPase activity-related genes were both significantly changed in the Δ*Ssrts1* mutants (Figure 8(b, c)). Heterotrimeric PP2A holoenzymes are known to coordinate complex spatially and temporally regulated functions within the cell, which requires a high degree of substrate specificity [51]. The regulatory subunits define the substrate specificity and the subcellular localization of the holoenzyme [52]. In yeast, PP2A-related protein phosphatase Sit4p-mediated dephosphorylation of Atp2p-T124/T317 downregulates Atp2p alongside with ATP synthase and mitochondrial respiration and ATP synthase [53]. According to the transcriptome analysis results, we speculate that the B regulatory subunit SsRts1 may interact with the Sit4 homolog SsPpe1 to cooperatively regulate ATPase activity. In addition, we found that there were more the number of DEGs in the JG36-Δ*Ssrts1* vs JG36 comparison than in the JG35-Δ*Ssrts1* vs JG35 comparison (Figure 7(a, c)). A similar phenomenon was also observed in our previous research. Furthermore, the same gene plays a different role in sexual mating among the different mating types strains, for example, *Ssppe1* [19]. It is known that there are differences in the mating-type loci between MAT-1 and MAT-2. Recent studies have found that the mating-type loci was different between SSC04 MAT-2 and SSC39 MAT-2 [54]. These results suggest that the difference number of DEGs in the JG36-Δ*Ssrts1* vs JG36 comparison than in the JG35-Δ*Ssrts1* vs JG35 comparison, may be due to the specific regulatory by SsRts1.

The Δ*Ssrts1* mutant strains exhibited more defects than the Δ*Ssppe1* mutant strains, including growth rate, sporidia morphology, mitosis and autophagy. The Δ*Ssppe1* mutants exhibited reduced pathogenicity and infectivity, whereas the Δ*Ssrts1* mutants completely lost its pathogenicity and infectivity. The more serious defects are determined by the modulation mechanisms of the regulatory subunits. Regulatory subunits can bind to different catalytic subunits to form numerous full-enzyme complexes. The regulatory subunits determine the substrate specificity and subcellular localization of the full-enzyme complexes, thereby coordinating the complex temporal regulation within the cell [51,52]. Therefore, regulatory subunits have a wider range of functions than catalytic subunits.

**Figure 10.**
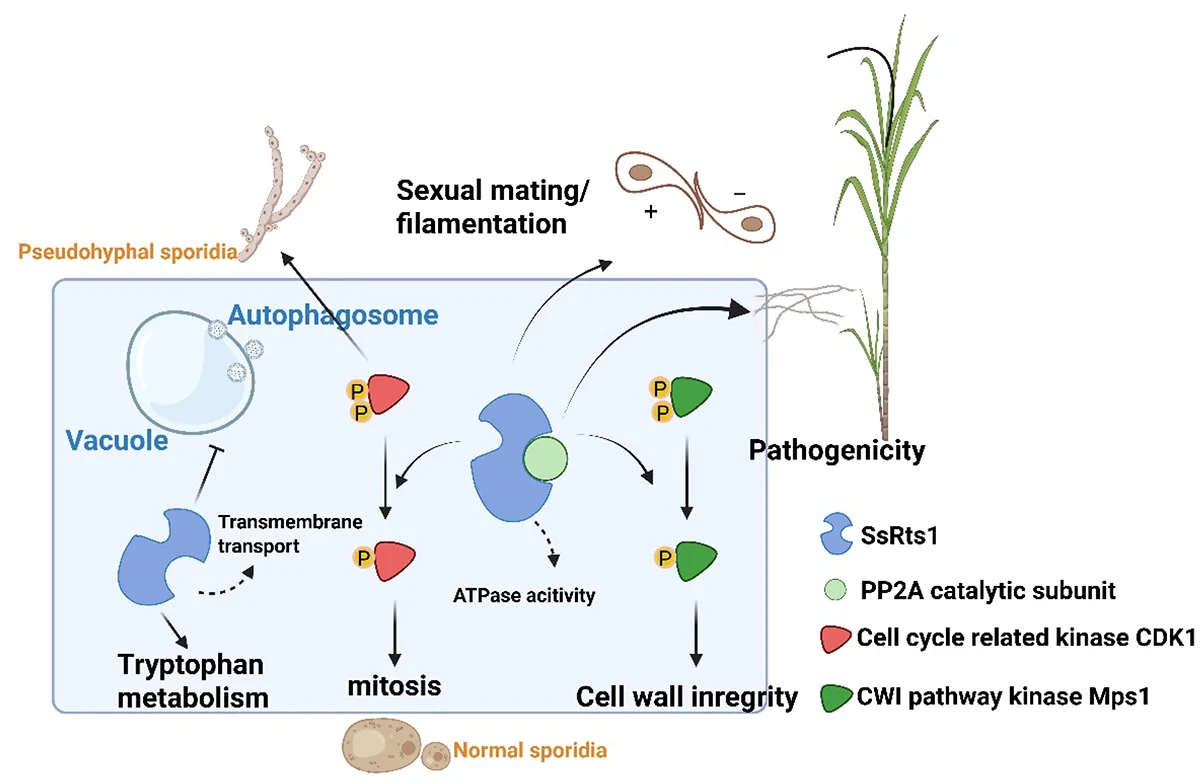
Working model showing the molecular mechanisms of SsRts1 in *S. scitamineum*. The PP2A regulatory subunit SsRts1 is crucial for mitosis, cell wall integrity, tryptophan metabolism, sexual mating/filamentation and pathogenicity.

A model summarizing our findings on this regulatory pathway is shown in Figure 10. In summary, this study provided evidence that PP2A regulatory subunit SsRts1 is involved in the regulation of cell wall integrity pathway, oxidative stress response and high osmotic pathway regulation, which are essential for mating, filamentous growth and virulence in *S. scitamineum*. In addition, we also revealed that SsRts1 can regulate the cell cycle, autophagy and tryptophan metabolism and ATPase activity. Together, the present findings provide a comprehensive insight into the function of the PP2A regulatory subunit Rts1 in filamentous fungi, and then expand the understanding of related studies.

## Experimental procedures

### Strains and culture conditions

The wild-type haploid strains of *S. scitamineum*, JG35 (MAT-2) and JG36 (MAT-1) were used in this study, which were previously isolated from smut-infected sugarcane plants in Guangxi province of China by our laboratory. The culture medium used in this study include YePSA plates or liquid YePS medium, MM medium. The protoplasts were regenerated on the 3-layer regeneration medium composed of top layer of YePS soft medium plus two layers of YePSS medium. The medium components are as previously described [19].

### cDNA library screen and yeast two-hybrid assays

The bait construct was generated by cloning SsPpe1 full-length cDNAs into the plasmid pGBKT7. After confirming the absence of autoactivation of the gene, Y2H screening was performed using a cDNA library constructed from *S. scitamineum*. We used the mating method to screen for the interact proteins of SsPpe1 [55]. To further confirm the interactions between SsPpe1 and SsRts1, the *Ssrts1* gene was cloned into the prey vector pGADT7 to generate a fusion protein as prey. The two constructs were co-transformed into the yeast strain Y2H and separately spread on the SD/-Leu-His and SD/-Leu-His-Trp-Ade medium. The plates were incubated at 28°C for 3 days, and photographed. The yeast co-transformation experiment was repeated at least three times.

### GST pull-down assay

GST pull-down assays were performed to validate protein–proteininteractions *in vitro*. The coding sequence of SsPpe1 was cloned into the pGEX-KG vector (Beijing Zoman Biotech, Beijing), in which SsPpe1 was fused with the GST tag at its N-terminal. The coding sequence of SsRts1 was cloned into the pET-32a vector to create a His-tagged fusion protein. All recombinant vectors were transformed into *Escherichia coli* strain BL21(DE3) competent cells for protein expression and purification. Fusion protein expression was induced with 1 mM IPTG at 16°C for 16 h. His-tagged and GST-tagged proteins were purified using Ni-NTA Resin (DP101-01, TransGen Biotech) and GST Resin (P2251, Beyotime), respectively. Equal amounts of the two purified proteins were mixed and incubated at 4°C for 8 h, followed by purification with GST Resin and collection of the bound complexes. Finally, input and output samples were analyzed by Western blot using anti-GST and anti-His antibodies, respectively. RealBand pre-stained protein marker II (C610110, Sangon Biotech) was used for molecular weight marker in Western blot. The co-incubation, purification and Western blotting experiments were repeated at least three times.

### Gene deletion and complementation

Gene deletion and complementation were performed using a split-marker strategy via polyethylene glycol (PEG)-mediated protoplast transformation, as previously described [59]. To delete the *Ssrts1* gene, the upstream and downstream sequences of the *Ssrts1* gene were PCR amplified from *S. scitamineum* genome DNA with primers *Ssrts1*-LB-F/R and *Ssrts1*-RB-F/R, respectively. The hygromycin-resistance gene (*HPT*) was amplified from plasmid pDAN with primers *HPT*-LB-F/226 and *HPT*-RB-225/R, respectively. The upstream and downstream flanking fragments (1,363 bp and 1,386 bp) were connected with partially fragments of *HPT* by fusion PCR, respectively. The two fusion homologous fragments were co-transformed into JG35 and JG36 protoplasts using PEG-mediated protoplast transformation. The putative deletion mutants were selected on YePS medium with 200 μg/mL hygromycin B and identified by PCR. Three independent transformants were generated per background.

For complementary strains, the complete *Ssrts1* gene, along with its native promoter (1,578 bp), was amplified by PCR with wild-type genome DNA as a template, using the primers PEASY-*Ssrts1*-F/R. The PCR products were ligated with the vector pEASY-COM. The *HPT*-LB and *HPT*-RB were two homologous upstream and downstream flanking fragments. Two fragments used for translation were PCR amplified with COM-LB-F/R and COM-RB-F/R primers, respectively. The two fragments were co-transformed into JG35-Δ*Ssrts1* and JG36-Δ*Ssrts1* protoplasts using PEG-mediated protoplast transformation. A zeocin-resistant cassette (*ZEO*^*R*^) was used as a selection marker by a split marker approach [59]. Putative complementation transformants were selected with 100 μg/mL zeocin and then confirmed by PCR. Primer pairs are listed in Table S1.

### Growth rate measurement

To assess growth rates, sporidia of the wild-type and the Δ*Ssrts1* mutants were inoculated into 5 mL YePS liquid medium and incubated at 28°C with shaking at 200 r min^−1^ for 24 h. The cultures were then diluted to OD_600_ = 0.1 with fresh YePS medium and incubated under the same conditions for 48 h. The sporidia suspension was measured by spectrophotometer (Bio-Rad) every 4 h to monitor growth. The experiment was conducted using three independent transformants.

### Microscopy

The morphology of the fungal colony was photographed using a Nikon SMZ25 stereomicroscope equipped with a digital camera. The haploid sporidia of WT and gene deletion strains were cultured to OD_600_ = 1.0. The YePS liquid medium was removed, and the sporidia were washed and resuspended in ddH_2_O. Staining solutions – including Calcofluor White (CFW), DAPI, neutral red, and monodansylcadaverine (MDC) – were then applied. A 10 μL aliquot of each sample was mounted on a glass slide and imaged using an Olympus BX51 microscope controlled by DP Controller software. Additionally, the cell wall of haploid sporidia was examined by transmission electron microscopy (TEM, JEOL, JEM-1400FLASH). The experiment was conducted with three independent transformants.

### Stress tolerance assay

The sporidia (1 μL, OD_600_ = 1.0) were spotted onto YePSA medium and MM-N medium supplemented with 0.5 M NaCl, 2.0 mM H_2_O_2_, 0.5 M Congo red, or 0.1 mM SDS after ten-fold serial dilutions, incubated at 28°C for 3 days, and photographed.

### Mating/filamentation assay

The haploid sporidia of opposite mating types (MAT-1 and MAT-2) were adjusted to OD_600_ = 1.0, mixed in equal volume and then spotted on the YePSA plates. The plates were incubated at 28°C for 3 days to observe the formation of dikaryotic hyphae. The experiment was conducted using three independent transformants and repeated at least three times.

### Analysis of protein phosphorylation levels

The fungal strains were ground and suspended in 1 mL protein extraction buffer (50 mM Tris, 0.5 mM EDTA, 50 Mm NaCl and 5% glycerol) and 10 µL phosphatase inhibitor cocktail (AR1183, BOSTER Biological Technology) and protease inhibitor mixture (P6730, Solarbio LIFE SCIENCE). After homogenization, the lysate was centrifuged at 12,000 rpm for 15 min at 4°C and the supernatant was collected. The total proteins were separated by 12% PAGE and then transferred to polyvinylidene fluoride (PVDF) membranes. The phosphorylation level of Mps1 was analyzed with phospho-p42/44 MAPK (ERK1/2) (Thr202/Tyr204) antibody (4370T, Cell Signaling Technology). The phosphorylation level of CDK1^T161^ was detected with phospho-CDK1-T161+CDK2/CDK3-T160 Rabbit mAb antibody (AP1466, Abclonal Technology). Actin was detected with β-Actin Rabbit mAb antibody (AC038, Abclonal Technology).

### RT-qPCR

Total RNA was extracted using TransZol (ET101-01, TransGen Biotech). The reverse transcription reaction was performed according to the protocol of HiScript III RT SuperMix for qPCR (R323-01, Vazyme), of which 900 ng RNA was used as the template. The RT-qPCR assay was conducted according to the instructions provided with the ChamQ SYBR qPCR Master Mix (Q711-02, Vazyme). For RT-qPCR analysis, three independent biological replicates were performed, with each biological replicate defined as independently strains. For each biological replicate, three technical replicates (parallel qPCR wells) were performed for each gene of interest to minimize pipetting errors. The relative gene expression level was calculated by adopting the 2^−ΔΔCt^ method, and the cytoskeletal protein gene *ACTIN* (*ACT1*) was used as an internal control [56]. All primers involved in construction, validation and RT-qPCR are listed in Table S1.

### Pathogenicity

The pathogenicity assay was performed using the soaking inoculation method as previously described [23]. We inoculated sugarcane with three independent transformants. The roots and bottom parts of the sugarcane tissue culture-derived plantlets were soaked in mixed sporidia suspension (OD_600_ = 1.0) and incubated at 28°C for three days. Subsequently, the plantlets were planted in nursery substrate and grown under natural conditions (28 ~ 35°C, 13-h/11-h light/dark photoperiod and relative air humidity of 80%~85%). Count the number of newly added black whips every five days, until no new black whips appear. Fungal colonization in the plant meristematic tissues was examined by staining with 0.4% trypan blue.

### Fungal biomass analysis

Sugarcane buds approximately 1–1.5 cm long were injected haploid sporidia mixture with a sterile needle, and then incubated under natural conditions (30 ~ 32°C, 13-h/11-h light/dark photoperiod and relative air humidity of 80%~85%). After 7 days, the total DNA was extracted from the buds using the FastPure Plant DNA Isolation Mini Kit (DC104, Vazyme). For the sugarcane with soaking inoculation, the total DNA was extracted from meristematic tissues approximately 1–1.5 cm long using same method. To quantify fungal biomass in sugarcane, an absolute quantification method using TaqMan qPCR was employed to determine the relative fungal-to-plant biomass ratio. The probes SsS11 and ScA5 were used to analyze the fungal and sugarcane biomass, respectively [57]. The reaction was conducted according to the instructions provided with the AceQ qPCR Probe Master Mix (Q112-02, Vazyme). Cope numbers were calculated using the standard curve formulas previously established in our laboratory [57].

### RNA-seq analysis

Total RNA was extracted from wild-type and Δ*Ssrts1* mutant strains, with three independent biological replicates performed for each comparison group. Construction of cDNA library, RNA sequencing and data analysis were conducted by Novogene company. RNA paired-end sequencing was performed on the Illumina NovaSeq 6000 in 2 × 150 bp runs. Raw reads were shown in the Table 2. Filter out adapter‑related reads, containing N reads and low-quality reads from raw reads yields clean reads [54], as shown in Table 2. Significant differential expression analysis between the wild-type and Δ*Ssrts1* mutant strains was performed using the DESeq2 package and identified with criteria *p* value ≤ 0.05 and |log_2_FoldChange| ≥1. For gene functional annotation, GO and KEGG enrichment analyses of DEGs were carried out using the clusterProfiler R package and the KEGG database (https://www.kegg.jp/kegg/) according to previous studies [58]. The threshold for enrichment was set as *p* value <0.05.

### Statistical analysis

All experiments in this study were performed with at least three independent biological replicates. Data are presented as mean ± *SE*. Statistical analysis was performed using GraphPad Prism 7 software, and *p* < 0.05 was considered statistically significant.

## Supplementary Material

Supplementary Material.docx

Table S1.docx

## Date availability statement

The RNA-Seq analysis raw date that support the findings of this study are available in NCBI Date accession with SRA BioProject number: PRJNA1393900. The source dates of this study are available in figshare with DOI number https://doi.org/10.6084/m9.figshare.30959663 (https://doi.org/10.6084/m9.figshare.30959663).
