# Supplementary material for: The PP2A phosphatase regulatory subunit SsRts1 regulates morphogenesis, mitosis, sexual mating and virulence in Sporisorium scitamineum
Source: Virulence. 2026 Aug 23;17(1):2723679. doi: 10.1080/21505594.2026.2723679 (PMC13531998; doi:10.1080/21505594.2026.2723679)
Supplement: Supplementary file 1 [file KVIR_A_2723679_SM0677.docx]

| **Table S1 Primers used in this study.** | | |
| --- | --- | --- |
| Primer | Sequence (5'-3') | Description |
| pGEX-*Ssppe1*-F | TCGGATCTGGTTCCGCGTGGATCCACCGTCGGCAATCCAGAT | Construction of pGEX-*Ssppe1* and pET32a-*Ssrts1* expression vector |
| pGEX-*Ssppe1*-R | ACCACCGGAAATTCCCGGGGATCCTCAGAGGAAGTACTGGCTATTC |  |
| pET32a-*Ssrts1*-F | GGCCATGGCTGATATCGGATCC ATGAAGGGCCTCAAGAAAG |  |
| pET32a-*Ssrts1*-R | CTCGAGTGCGGCCGCAAGCTT ACGTACCATATCGTCGACG |  |
| *HPT*-LB-F | AGATCTTGCTGATAGGCAGGTTTG | Construction of *Ssrts1* deletion mutants |
| *HPT*-RB-R | AGATCTAATTCGGGGGATCTGGAT |  |
| *HPT*-LB-226 | GGTCAAGACCAATGCGGAGC |  |
| *HPT*-RB-225 | GCAAGACCTGCCTGAAACCG |  |
| *Ssrts1*-LB-F | TGGCTACGTCAATCAAGGC |  |
| *Ssrts1*-LB-R | GTCGTGACTGGGAAAACCCTG GATGAATACGCGGCCGAG |  |
| *Ssrts1*-RB-F | GGTCATAGCTGTTTCCTGTGTGA TTCCGATGGAGAATGCTGG |  |
| *Ssrts1*-RB-R | TCGAGCGAGAGAGATGAA |  |
| *Ssrts1*-NY-F | CGACAACCACAGAACCTT |  |
| *Ssrts1*-NY-R | TCGCAGATTGAGGAACTT |  |
| *Ssrts1*-LBJC-F | ACACACACACACACACAC |  |
| *Ssrts1*-RBJC-R | TCGTGAAGGCGATAGCGT |  |
| *HPT*-F | GGCTCCAACAATGTCCTG |  |
| *HPT*-R | GTCAGGACATTGTTGGAG |  |
| PEASY-*Ssrts1*-F | GCGCCGATGGTTGATAGTTTAAAC CGCGATTCTAGTCTGTCG | Construction of *Ssrts1* complemention mutants |
| PEASY-*Ssrts1*-R | TCGTTTCCCGCCTTCAGTTTAAAC TCAACGTACCATATCGTCG |  |
| COM-LB-F | CCTCGAGCGATCCTTGAAGC |  |
| COM-LB-R | AGCGGGCAGTTCGGTTTCA |  |
| COM-RB-F | CAAGAACAAGCGCTGTCGCC |  |
| COM-RB-R | CGAGCATTCACTAGGCAACCA |  |
| zeo-F | ATGGCCAAGTTGACCAGT |  |
| zeo-R | TCAGTCCTGCTCCTCGGC |  |
| qRT-SsUAC1-F | GCACGACAACCTGAGTGTAA | qRT-PCR |
| qRT-SsUAC1-R | GCCGTCAAAGAGACCAAAGA |  |
| qRT-SsADR1-F | CGTGCTGCTCTACGAAATGC |  |
| qRT-SsADR1-R | AAGATCCTTGACGCCCGTTT |  |
| qRT-SsFUZ7-F | GCTCATCAACAGCATCGCAG |  |
| qRT-SsFUZ7-R | TCCGAATCGTCGTCGTCATC |  |
| qRT-SsCRK1-F | ACAGCCGAGCATCATCAACA |  |
| qRT-SsCRK1-R | GATGCAATGATGGCAGCTGG |  |
| qRT-SsKPP2-F | GATTGTCGATGTCGTTGGCG |  |
| qRT-SsKPP2-R | GGGCTTGACAATGTCGAGGA |  |
| qRT-SsROP1-F | TTCCCACCGTCTTCTGCATC |  |
| qRT-SsROP1-R | GTGGCTGCGAATATTGGCTG |  |
| qRT-SsPRF1-F | CAAGCAGTGTCACCGTTAGA |  |
| qRT-SsPRF1-R | GGAGAGCAAGGATGCAAGAT |  |
| qRT-SsPRA1-F | CCACGTAACACCTTTCTTTGCGC |  |
| qRT-SsPRA1-R | TCAATTCGCAATAACCCGGAGC |  |
| qRT-SsPRA2-F | AACGCTTCTTTCGGTGTTTTGAGC |  |
| qRT-SsPRA2-R | ATCGCAGCCGAAAGTCCATGTC |  |
| qRT-SsbW1-F | ATGTCGACCACTGTTCTATCTACTC |  |
| qRT-SsbW1-R | AATTTGTGAAGGTATAGGAGTCG |  |
| qRT-SsbW2-F | ATGTCAGCCTTTAACTCGTCCATAC |  |
| qRT-SsbW2-R | TACGCGTCGAGGTATGCAAAACTG |  |
| qRT-SsbE1-F | ATGGCGCAACACAGTAGCTTCGAG |  |
| qRT-SsbE1-R | CAGCTTGCCGTGCAAAGATGGTTGTC |  |
| qRT-SsbE2-F | GCCGACCAACAGCTTCCGATTC |  |
| qRT-SsbE2-R | GCAGCTTGACTTGCTTCGAACACC |  |
| qRT-SsMFA1-F | ATGCTTTCCATCTTTACCCAGA |  |
| qRT-SsMFA1-R | GTGCAGCTAGAGTAGCCAAG |  |
| qRT-SsMFA2-F | CGTCCAGGCCATTGTTTCT |  |
| qRT-SsMFA2-R | TAGGCCACGGTGCAGTA |  |
| qRT-ACTIN-F | CAGCTCGATGAAGGTCAAGAT |  |
| qRT-ACTIN-R | CACATCTGCTGGAAGGTAGAG |  |
| qRT-SsMKK1-F | CATCGCGACATCAAGCCAAG |  |
| qRT-SsMKK1-R | AGCTTGTACCCGTGAAGGTG |  |
| qRT-SsRHO1-F | CAGGGGACCAAGCCAGTATC |  |
| qRT-SsRHO1-R | CTACACGCCTCCCTAATCGC |  |
| qRT-SsRHO2-F | GAGGCAACAAGCCAAACAGG |  |
| qRT-SsRHO2-R | GCGTCGTTGTGTAATGCCTC |  |
| qRT-SsRHO3-F | ACGGTAGAGTTGACGCTGTG |  |
| qRT-SsRHO3-R | CCGGGTTATCGGTGGAGAAG |  |
| qRT-SsRac1-F | TCTGCTTTTCGCTCGTCAGT |  |
| qRT-SsRac1-R | CGATGGTCTCTGGGTCTTCG |  |
| qRT-SsCdc42-F | AGACCATCAAGTGCGTCGTT |  |
| qRT-SsCdc42-R | AATCCGAGCGTGTATGGGTC |  |
