# Supplementary material for: The PP2A phosphatase regulatory subunit SsRts1 regulates morphogenesis, mitosis, sexual mating and virulence in Sporisorium scitamineum
Source: Virulence. 2026 Aug 23;17(1):2723679. doi: 10.1080/21505594.2026.2723679 (PMC13531998; doi:10.1080/21505594.2026.2723679)
Supplement: Supplementary file 2 [file KVIR_A_2723679_SM0678.docx]

FIGURE S1. Construction and verification of *Ssrts1* deletion mutants. (a, b, c) PCR verification of *Ssrts1* genes, *HPT* left and right fragments in Δ*Ssrts1* mutants, respectively.

FIGURE S2. PCR verification of *Ssrts1* genes (a) and zeocin-resistant gene (b) in Δ*Ssrts1* and C-Δ*Ssrts1* mutants, respectively.

FIGURE S3. Expression analysis of Rho GTPase family genes by qRT-PCR in WT and Δ*Ssrts1* mutant strains. Error bars represent standard error (*n*=3), ****p*＜0.001.

FIGURE S4. Comparisons of Mps1 phosphorylation among wild-type, Δ*Ssrts1* and C-Δ*Ssrts1* strains. Western blots were performed with total proteins from sporidia of wild-type, Δ*Ssrts1* and C-Δ*Ssrts1* strains. The signal corresponding to phosphorylated Mps1 was detected by binding of the antiphospho-p42/44 antibody with the Actin antibody as a control. The ImageJ software was used to calculate the grayscale values. P-Mps1/Actin represents the ratio of the grayscale values of two bands.

FIGURE S5. The sexual mating behavior of the three Δ*Ssrts1* mutant strains. Wild-type strains and *Ssrts1* deletion mutants were co-spotted on YePSA plates and incubated at 28℃ for 3 days. The appearance of white, fluffy dikaryotic hyphae indicated a successful mating reaction.

FIGURE S6. Confirmation of the expression of 10 randomly chosen DEGs by RT-qPCR analysis between the JG35 vs. JG35-Δ*Ssrts1* strains (a) and JG36 vs. JG36-Δ*Ssrts1* strains (b).

FIGURE S7. Representative images show that exogenous supplementation of Trp (50, 100 and 150 µM) cannot restore the filamentous growth defect of the Δ*Ssrts1* mutants.
